# Quantitative Proteome Analysis of *Atg5*-Deficient Mouse Embryonic Fibroblasts Reveals the Range of the Autophagy-Modulated Basal Cellular Proteome

**DOI:** 10.1128/mSystems.00481-19

**Published:** 2019-11-05

**Authors:** Kiran Bala Sharma, Manish Sharma, Suruchi Aggarwal, Amit Kumar Yadav, Shinjini Bhatnagar, Sudhanshu Vrati, Manjula Kalia

**Affiliations:** a Translational Health Science and Technology Institute, NCR Biotech Science Cluster, Faridabad, Haryana, India; b Regional Centre for Biotechnology, NCR Biotech Science Cluster, Faridabad, Haryana, India; Princeton University

**Keywords:** *Atg5*, IL-6, JAK-STAT, TLR2, TMT mass spectrometry, cell adhesion, cytokine receptors, inflammation, innate immune response, interferon

## Abstract

Basal autophagy is crucial for maintenance of cellular homeostasis. ATG5 is an essential protein for autophagosome formation, and its depletion has been extensively used as a tool to disrupt autophagy. Here, we characterize the impact of *Atg5* deficiency on the cellular proteome of mouse embryonic fibroblasts (MEFs). Using a tandem mass tagging (TMT)-based quantitative proteomics analysis, we observe that 14% of identified proteins show dysregulated levels in *atg5^−/−^* MEFs. These proteins were distributed across diverse biological processes, such as cell adhesion, development, differentiation, transport, metabolism, and immune responses. Several of the upregulated proteins were receptors involved in transforming growth factor β (TGF-β) signaling, JAK-STAT signaling, junction adhesion, and interferon/cytokine-receptor interactions and were validated as autophagy substrates. Nearly equal numbers of proteins, including several lysosomal proteins and enzymes, were downregulated, suggesting a complex role of autophagy/ATG5 in regulating their levels. The *atg5^−/−^* MEFs had lower levels of key immune sensors and effectors, including Toll-like receptor 2 (TLR2), interferon regulatory factor 3 (IRF3), IRF7, MLKL, and STAT1/3/5/6, which were restored by reexpression of ATG5. While these cells could efficiently mount a type I interferon response to the double-stranded RNA (dsRNA) mimic poly(I·C), they were compromised in their inflammatory response to the bacterial pathogen-associated molecular patterns (PAMPs) lipopolysaccharide (LPS) and Pam3CSK4. Transcriptional activation and secretion of interleukin-6 (IL-6) in these cells could be recovered by ATG5 expression, supporting the role of autophagy in the TLR2-induced inflammatory response. This study provides a key resource for understanding the effect of autophagy/ATG5 deficiency on the fibroblast proteome.

**IMPORTANCE** Autophagy performs housekeeping functions for cells and maintains a functional mode by degrading damaged proteins and organelles and providing energy under starvation conditions. The process is tightly regulated by the evolutionarily conserved *Atg* genes, of which *Atg5* is one such crucial mediator. Here, we have done a comprehensive quantitative proteome analysis of mouse embryonic fibroblasts that lack a functional autophagy pathway (*Atg5* knockout). We observe that 14% of the identified cellular proteome is remodeled, and several proteins distributed across diverse cellular processes with functions in signaling, cell adhesion, development, and immunity show either higher or lower levels under autophagy-deficient conditions. These cells have lower levels of crucial immune proteins that are required to mount a protective inflammatory response. This study will serve as a valuable resource to determine the role of autophagy in modulating specific protein levels in cells.

**Author Video**: An author video summary of this article is available.

## Abstract (precis)

Autophagy performs housekeeping functions for cells and maintains a functional mode by degrading damaged proteins and organelles and providing energy under starvation conditions. The process is tightly regulated by the evolutionarily conserved *Atg* genes, of which *Atg5* is one such crucial mediator. Here, we have done a comprehensive quantitative proteome analysis of mouse embryonic fibroblasts that lack a functional autophagy pathway (*Atg5* knockout). We observe that 14% of the identified cellular proteome is remodeled, and several proteins distributed across diverse cellular processes with functions in signaling, cell adhesion, development, and immunity show either higher or lower levels under autophagy-deficient conditions. These cells have lower levels of crucial immune proteins that are required to mount a protective inflammatory response. This study will serve as a valuable resource to determine the role of autophagy in modulating specific protein levels in cells.

## INTRODUCTION

Macroautophagy (here autophagy) acts as a housekeeping module to keep the cellular system clean by constitutively maintaining protein turnover and removing damaged organelles and aggregated proteins. During starvation, it turns into a “lifeguard” to provide survival energy by degrading the nonessential components of the cell. Autophagy targets a diverse range of substrates and hence is involved in the regulation of several cellular pathways impacting development, metabolism, signal transduction, aging, and immune function. Autophagy also plays critical roles under diverse conditions such as pathogen infection, cancer, and neurodegeneration (1–3).

Autophagosome biogenesis is executed by the action of the evolutionarily conserved ATG genes (4). In response to signals such as mTOR inhibition, phagophore formation is initiated by the ULK1-ATG3-FIP200 complex and the class III phosphatidylinositol 3-kinase (PI3-K)-Beclin1 complex (5, 6). Elongation of the phagophore involves two ubiquitin-like (UBL) conjugation systems. A series of enzymatic events leads to the activation and formation of the ATG12-ATG5-ATG16L1 molecular complex that binds to the phagophore and completes the loading of phosphatidylethanolamine (PE) conjugated to microtubule-associated protein 1 light chain 3 (LC3) on the inner and outer membranes of the autophagosome (7, 8). Given its central and crucial role in autophagosome formation, ATG5 depletion has been widely used as a powerful tool to understand autophagy and processes regulated by it, at both the cellular and organism levels (9–21).

In recent years, several technical advances in mass spectrometry have enabled an enhanced capacity for proteomic discovery (22). High-throughput quantitative proteomics is a highly sensitive approach to analyze global protein dynamics within a cell (23). By directly focusing on the biological effector molecules, it provides several advantages over mRNA expression analysis. Here, we present a tandem mass tagging (TMT)-based mass spectrometry analysis of wild-type (WT) and *atg5^−/−^* mouse embryonic fibroblasts (MEFs) and analyze the role of autophagy/ATG5 in remodeling the cellular fibroblast proteome under basal conditions. We observe that 14% of the cellular proteome is dysregulated due to ATG5 deficiency, and proteins implicated in diverse biological functions, such as development, cell adhesion, signal transduction, metabolism, and immune and inflammatory processes, are impacted. Several of the upregulated proteins were receptors, indicating an important role of basal autophagy in the constitutive turnover of receptors. Multilayered cellular regulation through ATG5 could also be seen, as equal numbers of proteins were downregulated in these cells. Several of the downregulated proteins were critical modulators of immune and inflammatory responses. The *atg5^−/−^* MEFs expressed low levels of Toll-like receptor 2 (TLR2) and were inefficient in mounting a response to the bacterial pathogen-associated molecular patterns (PAMPs) lipopolysaccharide (LPS) and Pam3CSK4, and this effect was reversed by reexpression of ATG5. This study will serve as a useful proteomic resource for the impact of autophagy deficiency in fibroblasts.

## RESULTS

### TMT-based mass spectrometry of WT and *atg5^−/−^* MEFs.

**FIG 1:**
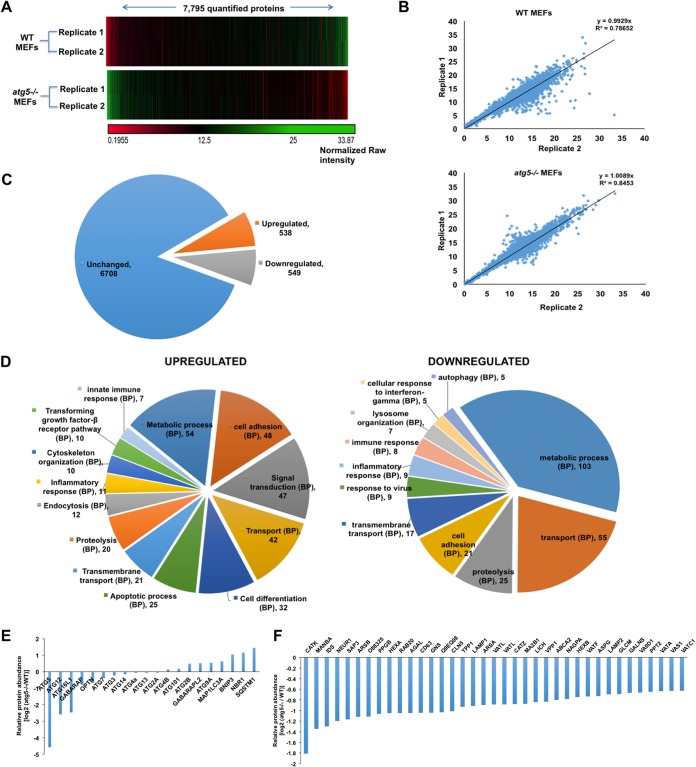
TMT-based mass spectrometry analysis of WT and *atg5^−/−^* MEFs. (A) Hierarchical cluster heat map showing the levels of 7,795 (≥2 unique peptides and <1% FDR) quantified proteins across the WT and *atg5^−/−^* MEFs. The normalized raw intensities were used for creating the heat map using the Gene-E tool. (B) Scatterplot displaying the relative protein abundances of 7,795 proteins in the sample duplicates of WT and *atg5^−/−^* MEFs. Pearson’s correlation is indicative of the reproducibility of the biological replicates. (C) Fold change (*atg5^−/−^*/WT) intensities of the 7,795 quantified proteins. The pie chart displays the numbers of unchanged, upregulated (≥1.5-fold), and downregulated (≤1.5-fold) proteins. (D) Gene ontology (GO) enrichment analysis of upregulated and downregulated proteins in *atg5^−/−^* MEFs was performed using GeneCodis to study biological processes (BP). (E and F) Bar graphs showing the normalized relative protein abundances of several ATG proteins and autophagy substrates (E) and of different proteins involved in lysosomal organization and pH regulation (F) in *atg5^−/−^* MEFs.

To study the impact of basal autophagy on the cellular proteome, we analyzed results of a TMT-based mass spectrometry quantitative proteomics study for biological replicates of WT and *atg5^−/−^* MEFs (see Fig. S1 in the supplemental material). A total of 8,745 proteins were quantified from each sample (1% false discovery rate [FDR]). Proteins having fewer than 2 unique peptides were excluded, and the resulting 7,795 proteins were used for further analysis. A hierarchical cluster heat map shows changes in relative protein abundances across the WT and ATG5-deficient conditions (Fig. 1A). A comparison of the replicates showed good correlation between the sample duplicates of WT and *atg5^−/−^* cells (Fig. 1B). Replicates were combined based on their normalized percent relative abundances, and the protein intensity fold change (FC) between the two cell types was calculated. We found that the absence of ATG5 affected ∼14% (1,087 of 7,795) of the identified proteome, with 538 (6.9%) proteins upregulated (≥1.5-fold) and 549 (7.04%) proteins downregulated (≤1.5-fold) in *atg5^−/−^* MEFs (Fig. 1C and Data Set S1).

10.1128/mSystems.00481-19.1 FIG S1 (A) Schematic of the TMT-based proteomics workflow. For the present study, biological replicates of the WT and *atg5^−/−^* (KO) MEFs (left) were analyzed. (B) The normalized raw intensities for all samples were used to create the principal component analysis score plots using the ClustVis server. Download FIG S1, TIF file, 0.5 MB. Copyright © 2019 Sharma et al. 2019 Sharma et al. This content is distributed under the terms of the Creative Commons Attribution 4.0 International license.

10.1128/mSystems.00481-19.7 DATA SET S1 Normalized percent relative abundances of 7,795 proteins in WT and *atg5*^−/−^ MEFs. Data are sorted based on protein intensity fold changes (sheet 1). Also shown are proteins differentially regulated under low (sheet 2)-, medium (sheet 3)-, and high (sheet 4)-stringency threshold conditions. Download Data Set S1, XLSX file, 4.7 MB. Copyright © 2019 Sharma et al. 2019 Sharma et al. This content is distributed under the terms of the Creative Commons Attribution 4.0 International license.

**FIG 2:**
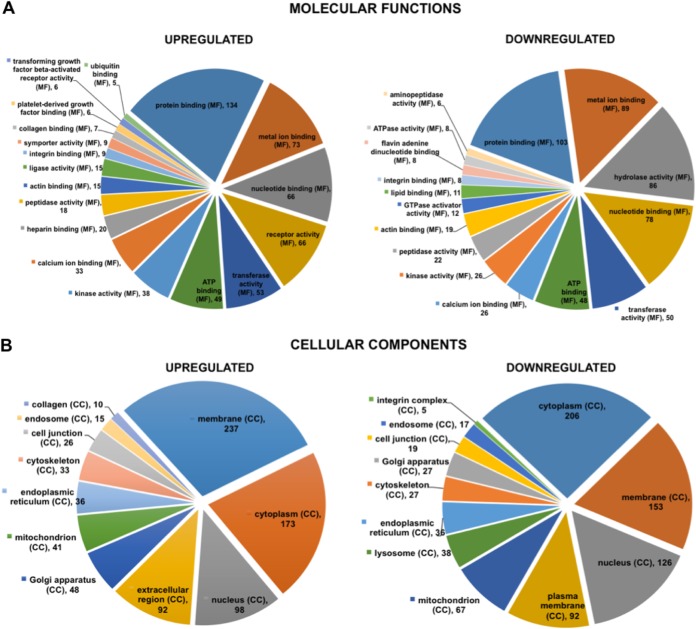
GO enrichment analysis of up- and downregulated proteins found in *atg5^−/−^* MEFs was performed using GeneCodis to identify molecular functions (MF) (A) and cellular components (CC) (B).

Pathway enrichment analysis was performed to segregate the dysregulated proteins based on biological processes, cellular components, and molecular functions (Fig. 1D, Fig. 2, and Data Set S2). We observed that several proteins implicated in cell adhesion, proteolysis, transport, metabolic processes, signal transduction, and immune and inflammatory responses were dysregulated in *atg5^−/−^* MEFs (Fig. 1D and Data Set S2). The proteins affected by autophagy modulation had diverse molecular functions, such as protein, lipid, metal ion, nucleotide, ATP binding, hydrolase, transferase, kinase, and peptidase activities, etc., and different subcellular localizations (Fig. 2 and Data Set S2).

10.1128/mSystems.00481-19.8 DATA SET S2 Gene ontology enrichment analysis (biological processes, cellular components, and molecular functions) of proteins up- and downregulated in *atg5^−/−^* MEFs. Download Data Set S2, XLSX file, 0.02 MB. Copyright © 2019 Sharma et al. 2019 Sharma et al. This content is distributed under the terms of the Creative Commons Attribution 4.0 International license.

The levels of ATG5, ATG12, and ATG16L1 were reduced by over 90% in *atg5^−/−^* MEFs. Other autophagy proteins, ATG3, ATG7, GABARAP, and OPTN, displayed 20 to 40% reductions in levels, while the levels of ATG2A, ATG4A, ATG4B, ATG13, and ATG14 were found to be unchanged (Fig. 1E). Consistent with the literature, we also observed the accumulation of autophagy substrates such as SQSTM1, NBR1, and BNIP3 in *atg5^−/−^* MEFs (Fig. 1E). These cells also had lower levels of lysosomal proteins, enzymes, and vacuolar ATPases, indicative of a reduced lysosomal compartment in these cells (Fig. 1F, Fig. 2B, Fig. S2, and Fig. S3B).

10.1128/mSystems.00481-19.2 FIG S2 Gene ontology (GO) enrichment analysis of proteins with potential LIR motifs (PSSM score of ≥13) was done using GeneCodis to identify KEGG pathways (A) and biological processes (B). Download FIG S2, TIF file, 0.6 MB. Copyright © 2019 Sharma et al. 2019 Sharma et al. This content is distributed under the terms of the Creative Commons Attribution 4.0 International license.

10.1128/mSystems.00481-19.3 FIG S3 KEGG analysis of cellular transporter proteins (A and B) and metabolism-related proteins (C and D) found to be differentially regulated in *atg5^−/−^* MEFs. Download FIG S3, TIF file, 0.7 MB. Copyright © 2019 Sharma et al. 2019 Sharma et al. This content is distributed under the terms of the Creative Commons Attribution 4.0 International license.

Several proteins implicated in cell differentiation, transport, signal transduction, cytoskeleton reorganization, endocytosis, apoptosis, and innate immune responses were selectively upregulated in *atg5^−/−^* MEFs (Fig. 1D). Molecular function and cellular compartment analyses showed that many of these proteins were receptors with an extracellular localization but diverse functions, such as the transforming growth factor β (TGF-β) receptor family; platelet-derived growth factor binding; collagen, ubiquitin, and heparin binding; and the low-density lipoprotein (LDL) receptor (LDLR) and LDL receptor-related protein families (Fig. S2A and B). Since all the proteins that have higher levels in *atg5^−/−^* MEFs are likely to be autophagy substrates, these were checked for potential LC3-interacting region (LIR) motifs, and the position-specific scoring matrix (PSSM) score was calculated (24). A total of 339 (63%) upregulated proteins had a PSSM score of >13, suggesting that these are potential autophagy substrates (Fig. S2 and Data Set S3).

10.1128/mSystems.00481-19.9 DATA SET S3 PSSM scores of all proteins upregulated in *atg5^−/−^* MEFs and their pathway enrichment analysis. Download Data Set S3, XLSX file, 0.1 MB. Copyright © 2019 Sharma et al. 2019 Sharma et al. This content is distributed under the terms of the Creative Commons Attribution 4.0 International license.

In the *atg5^−/−^* MEFs, several crucial transporters were also found to be dysregulated (Fig. S3A and B). Of these, proteins that function in membrane trafficking (LDLR, STX6, RAB39b, KDELR3, SYT11, and SNX17), the solute carrier (SLC) superfamily, the ABC transporter superfamily, and lipid transporters were selectively upregulated (Fig. S3A and B and Data Set S4), while other proteins, such as vacuolar ATPases, were downregulated (Fig. S3B and Data Set S4). In accordance with the role of autophagy in regulating cellular homeostasis, proteins implicated in multiple metabolic pathways (lipid, carbohydrate, sphingolipid, glutathione, ceramide, and amino sugar, etc.) were dysregulated (Fig. S3C and D and Data Set S4).

10.1128/mSystems.00481-19.10 DATA SET S4 List of transporters, metabolic pathway-associated proteins, development pathway-associated proteins, cell adhesion proteins, and immune-related proteins differentially expressed in *atg5^−/−^* MEFs manually annotated using KEGG, GeneCodis (biological function), and Reactome. Download Data Set S4, XLSX file, 0.02 MB. Copyright © 2019 Sharma et al. 2019 Sharma et al. This content is distributed under the terms of the Creative Commons Attribution 4.0 International license.

### Autophagy is a critical regulator of proteins involved in development.

Sonic Hedgehog, TGF-β, fibroblast growth factor (FGF), Notch, and Wnt signaling proteins are crucial for early patterning and organization in embryonic development and also control cell proliferation and differentiation throughout life (25). The literature has demonstrated the importance of autophagy in embryogenesis and development, as deletion of autophagy genes in mice leads to lethality (embryonic and neonatal stages) and defects in neuronal differentiation (26, 27). Several studies have shown the interplay between autophagy/autophagy-related genes (ATGs) and developmental pathways (Wnt, Notch, Shh, FGF, and TGF-β) in different cell models (28–33). Autophagy balances the levels of various development-related signaling proteins. It is well known to antagonize Wnt signaling by degrading disheveled protein in autolysosomes in normal rat kidney epithelial cells and MEFs (28, 30) and by eliminating cytoplasmic beta-catenin (31, 34), Notch signaling by degrading Notch receptor and controlling neurogenesis and stem cell growth (33), and TGF-β signaling by degrading TGF-β (29) and TGF-β receptor 1 (TGF-βR1) (35).

**FIG 3:**
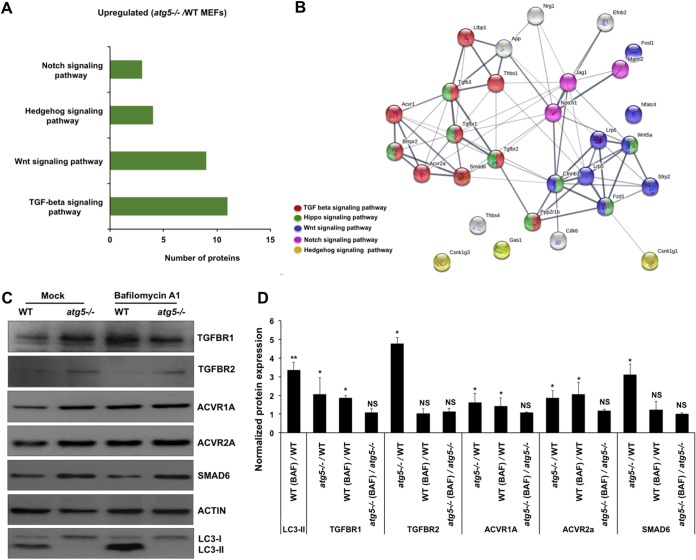
Autophagy is a critical regulator of proteins involved in development. (A) KEGG pathway analysis of the upregulated development-related proteins in *atg5^−/−^* MEFs was performed using GeneCodis. (B) A functional protein association network was generated using STRING 11.0. The line thickness indicates the strength of data support. (C) WT and *atg5^−/−^* MEFs were treated with the vehicle control (dimethyl sulfoxide [DMSO]) or 100 nM bafilomycin A_1_ (Baf A_1_) for 3 h. Protein lysates were analyzed by Western blotting with TGF-βR1, TGF-βR2, ACVR1A, ACVR2A, SMAD6, LC3, and actin (loading control) antibodies. (D) Bar graph showing normalized protein levels in KO MEFs (*atg5^−/−^/*WT), Baf A_1_-treated WT MEFs [WT (Baf)/WT], and Baf A_1_-treated KO MEFs [*atg5^−/−^*(Baf)/*atg5^−/−^*]. An increase in the protein level upon Baf A_1_ treatment as seen for LC3-II is indicative of protein degradation through autophagy. Densitometry analysis of the protein bands was performed using ImageJ software. Data are presented as means ± SD of values obtained from 3 independent experiments. Student’s *t* test was used to calculate *P* values (*, *P < *0.05; **, *P < *0.01; NS, not significant).

In accordance with published data, *atg5^−/−^* MEFs showed enhanced levels of key players involved in development and differentiation pathways such as TGF-β, Wnt, Hedgehog, and Notch (Fig. 3A and B and Data Set S4). We validated the higher expression levels of some of the key molecules of the TGF-β signaling pathway in *atg5^−/−^* MEFs by Western blotting. Significantly higher levels of TGF-βR1, TGFβ-R2, ACVR1A, ACVR2A (activin A receptors of the bone morphogenetic protein [BMP] pathway), and SMAD6 were observed (Fig. 3C and D). SMAD6 is an inhibitory SMAD that binds and inactivates phosphorylated receptor SMADs, preventing nuclear translocation and transcription of genes that promote osteoblast differentiation (36). SMAD6 also inhibits BMP signaling by complexing with the ubiquitin ligase SMURF1, leading to the proteasomal degradation of BMP receptors and receptor SMADs (37).

To distinguish the protein accumulations caused by ATG5 deficiency versus a block in autophagosome degradation, we used the vacuolar ATPase inhibitor bafilomycin A_1_ (Baf A_1_), which inhibits vesicle acidification and thus prevents autophagosome maturation into autolysosomes and subsequent degradation of cargo. As expected, treatment of WT MEFs with Baf A_1_ increased levels of LC3-II significantly (Fig. 3C and D). A similar increase in protein levels of TGF-βR1, ACVR1A, and ACVR2A was observed in WT MEFs upon Baf A_1_ treatment, validating that autophagy is involved in the turnover of these receptors in the cell (Fig. 3C and D). The relative mRNA levels of a subset of TGF-β signaling pathway genes (*Tgf*β*r1*, *Tgf*β*r2*, *Tgf*β*r3*, *Acvr1a*, *Acvr2a*, *Smad6*, and *Bmpr2*) were also evaluated (Fig. S4A). While levels of some transcripts were unchanged, significantly lower levels of *Tgf*β*r1* and higher levels of *Tgf*β*r2* and *Tgf*β*r3* were observed in *atg5^−/−^* MEFs. This was not unexpected since protein levels are influenced by multiple factors and do not always correlate with mRNA levels. Collectively, our data confirm the involvement of autophagy in regulating the expression of key TGF-β receptor signaling components.

10.1128/mSystems.00481-19.4 FIG S4 mRNA levels of TGF-β receptor signaling genes (A), cell adhesion-related genes (B), and immune-related genes (C) in *atg5^−/−^* MEFs normalized to values for WT MEFs, determined by quantitative qRT-PCR. Values represent means and SD of data from 3 independent experiments. Download FIG S4, TIF file, 0.4 MB. Copyright © 2019 Sharma et al. 2019 Sharma et al. This content is distributed under the terms of the Creative Commons Attribution 4.0 International license.

### Autophagy regulates levels of several cell adhesion proteins.

**FIG 4:**
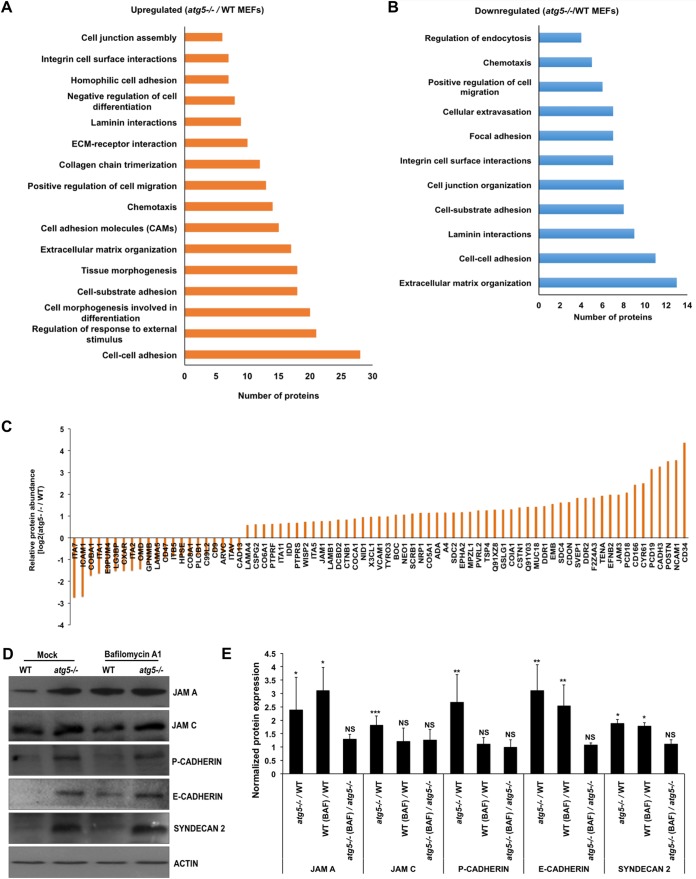
ATG5 deficiency dysregulates several cell adhesion proteins. (A and B) KEGG analysis of cell adhesion proteins found to be up- and downregulated upon autophagy deficiency. (C) Bar graph showing the differential expression of integrin and collagen proteins due to autophagy deficiency. (D) WT and *atg5^−/−^* MEFs were treated with DMSO (control) and 100 nM Baf A_1_ for 3 h, and protein extracts were analyzed by Western blotting with JAMA, JAMC, P-cadherin, E-cadherin, syndecan 2, and actin (loading control) antibodies. (E) Bar graph showing the normalized protein levels in KO MEFs (*atg5^−/−^*/WT), Baf A_1_-treated WT MEFs [WT (Baf)/WT], and Baf A_1_-treated KO MEFs [*atg5^−/−^*(Baf)/*atg5^−/−^*]. An increase in the protein level upon Baf A_1_ treatment is indicative of protein degradation through autophagy. Densitometry analysis was performed using ImageJ software. Presented are means ± standard deviations of values obtained from 3 independent experiments. Student’s *t* test was used to calculate *P* values (*, *P < *0.05; **, *P < *0.01; ***, *P < *0.001).

Cell adhesion emerged as a major biological process whose proteins were dysregulated in *atg5^−/−^* MEFs (Fig. 4A and B and Data Set S4). Several studies have documented an essential role of autophagy in establishing tight junction permeability, cell adhesion, cell motility, and tumor metastasis (38, 39). The turnover of cadherin (40, 41), Claudin2 (42), internalized collagen and focal adhesion (FA) proteins (43, 44), VCAM1 in endothelial cells (45), cytokine-induced ICAM1 levels in lung epithelial cells (46), and the actin cytoskeleton and adherens junctions in human induced pluripotent stem cells (47) are known to be regulated by autophagy.

In our data, we found that the absence of autophagy/ATG5 altered the expression levels of numerous cell adhesion molecules that are essential for focal adhesion, adherens junctions, tight junctions, extracellular matrix-receptor interactions, and leukocyte transendothelial migration in the cells (Fig. 4A and B). Levels of adhesion proteins, including members of the immunoglobulin superfamily (JAM1, JAM3, VCAM1, and NCAM1), cadherins (CDH3, PCDHB22, PCDH19, PCDH18, and PCDH16), integrins (ITGA5 and ITGA11), syndecans (SDC2 and SDC4), laminins (LAMA4 and LAMB1), and collagens (COL6a1, COL5a1, COL12a1, and COL18a1), were found to be increased, whereas levels of proteins such as CDH13, LAMA5, ICAM1, integrins (ITGAV, ITGA2, ITGA1, ITGB5, and ITGA7), and collagens (COL8a1 and COL11a1) were found to be decreased in ATG5-deficient MEFs (Fig. 4C and Data Set S4).

We validated the expression levels of some of these proteins by Western blotting and observed significantly higher levels of JAM1, JAM3, P-cadherin, E-cadherin, and SDC2 in autophagy-defective cells than in WT cells (Fig. 4D and E). WT and *atg5^−/−^* MEFs were also treated with Baf A_1_, and cell lysates were further analyzed by Western blotting. In WT MEFs, Baf A_1_ treatment led to a rapid accumulation of LC3-II, indicating that autophagosomes are not being turned over by lysosomal proteolysis, whereas, as expected, *atg5^−/−^* MEFs did not show LC3-II (Fig. 3C and D). WT MEFs showed an enhancement of JAM1, E-cadherin, and SDC2 levels in Baf A_1_-treated samples, while the intensity of JAM3 and P-cadherin remain unchanged (Fig. 4D and E). Several of these proteins were also transcriptionally upregulated in *atg5^−/−^* MEFs (Fig. S4B). Our data thus indicate that ATG5/autophagy plays a major role in modulating levels of proteins involved in cell adhesion, motility, and cell communication.

### Absence of ATG5 alters levels of several immune effectors.

**FIG 5:**
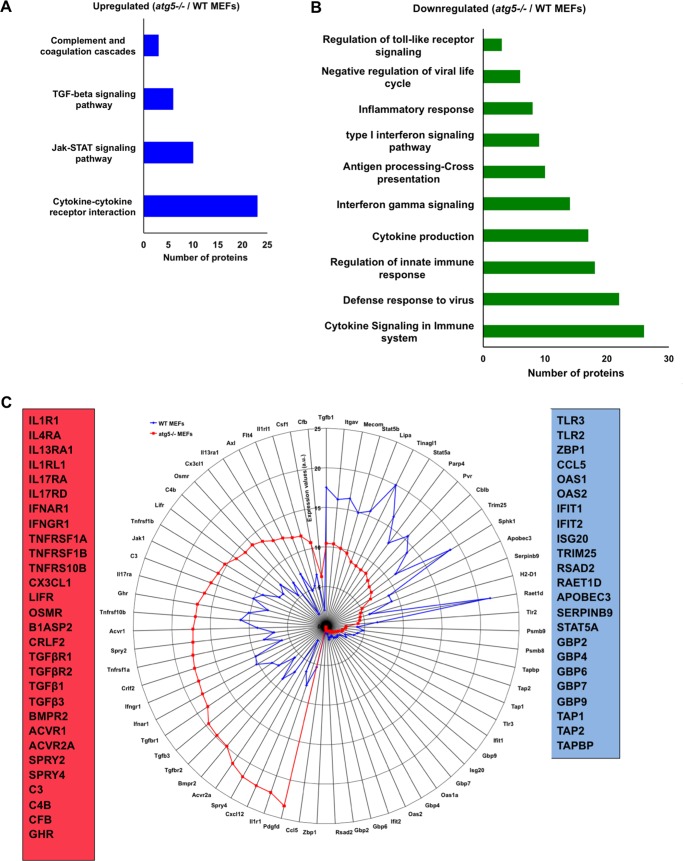
ATG5-deficient MEFs show dysregulation of several immune-related proteins. (A and B) KEGG analysis of immune-related proteins found to be up- and downregulated due to autophagy deficiency. (C) Radar plot representing the expression values of the listed immune proteins in WT and *atg5^−/−^* MEFs.

Our proteome analysis showed differential expression patterns for various immune-related proteins, suggesting that autophagy/ATG5 may be crucial in regulating interferon (IFN) and inflammatory and adaptive immune responses. We observed elevated levels of several cytokine receptors, interferon receptors, tumor necrosis factor (TNF) receptors, TGF receptors, and complement system proteins in *atg5^−/−^* MEFs (Fig. 5A to C and Data Set S4). Studies have shown that the IFNAR1 and IFNGR1 receptors undergo ubiquitin-dependent lysosomal degradation (48–50). Similarly, plasma membrane levels of TNFR1 and LIFR are maintained via endocytosis and degradation in lysosomes (51, 52). The complement protein C3 directly interacts with ATG16L1 and is involved in autophagy-dependent bacterial growth restriction and recycling in diabetogenic stress (53, 54). Several of these proteins also have LIR motifs (Fig. S2 and Data Set S3), suggesting that autophagy might be engaged in maintaining the basal turnover of these immune signaling receptors by degradation in lysosomes. Furthermore, *atg5^−/−^* MEFs also have higher protein levels of JAK1, a protein noncovalently associated with the cytoplasmic tail of several cytokine receptors, including IFNAR and IFNGR (55).

On the other hand, many proteins known to play a fundamental role in pathogen recognition (TLR2, TLR3, and ZBP1) and the activation of innate (OAS1, OAS2, IFIT1, IFIT2, TRIM25, ISG20, and the GBP family) and adaptive (HLA-A, TAP1, TAP2, TAPBP, PSMB8, and PSMB9) immunity were suppressed in autophagy-deficient cells (Fig. 5B and C and Data Set S4). This is indicative of a strong link between basal autophagy/ATG5 and levels of immune proteins in resting cells.

**FIG 6:**
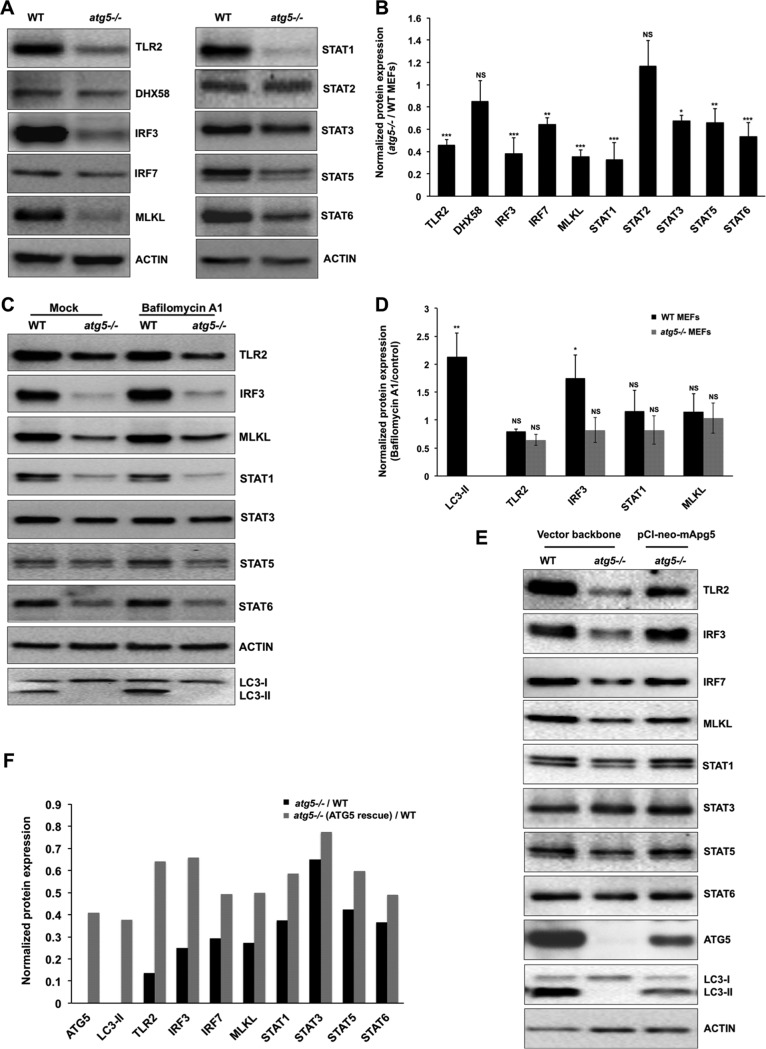
ATG5-deficient MEFs have lower levels of immune signaling proteins and STATs, which can be rescued by ATG5 reexpression. (A and B) Western blots and bar graphs showing levels of TLR2, DHX58, IRF3, IRF7, MLKL, STAT1, STAT2, STAT3, STAT5, STAT6, and actin (loading control) in WT and *atg5^−/−^* MEFs. (C and D) WT and *atg5^−/−^* MEFs were treated with DMSO (control) or 100 nM Baf A_1_ for 3 h, and protein extracts were analyzed by Western blotting. The Western blots and bar graphs show the levels of the indicated proteins. (E and F) ATG5 expression in *atg5^−/−^* MEFs was performed by transfection with a vector backbone or pCI-neo-mApg5. For panels B and D, data in bar graphs showing normalized protein levels (*atg5^−/−^*/WT [B] and Baf A_1_/mock [D]) are presented as means ± SD of values obtained from 3 independent experiments. (F) Bar graphs showing normalized protein levels (*atg5^−/−^*/WT and ATG5 rescue/WT) presented as mean values obtained from 2 independent experiments. Densitometry analysis was done using ImageJ software. Student’s *t* test was used to calculate *P* values (*, *P < *0.05; **, *P < *0.01; ***, *P < *0.001).

Using Western blotting, we validated the expression levels of some crucial innate immune-related and STAT family proteins in *atg5^−/−^* MEFs (Fig. 6). Significant reductions in the levels of TLR2, interferon regulatory factor 3 (IRF3), IRF7, MLKL, STAT1, STAT3, STAT5, and STAT6 was observed in these cells (Fig. 6A and B). The transcriptional levels of these genes in both cell lines showed significant downregulation of *Irf7*, *Mlkl*, *Stat1*, and *Stat5* in *atg5^−/−^* MEFs (Fig. 6C). We further treated WT and *atg5^−/−^* MEFs with Baf A_1_ to distinguish innate immune effector suppression caused by autophagy degradation impairment versus ATG5 absence. No change in TLR2, MLKL, and STAT1 levels upon Baf A_1_ treatment in WT MEFs was observed. However, levels of the primary innate immune effector IRF3 showed an increase, implying that its basal levels are maintained through autophagy (Fig. 6C and D). To validate that the lower protein levels of these key immune sensors and adaptors was a direct effect of ATG5 deficiency, we overexpressed ATG5 in the *atg5^−/−^* MEFs (Fig. 6E and F). Expression of ATG5 and conversion of LC3-I to LC3-II were seen in these cells, confirming restoration of autophagy function. The levels of all the key immune-related proteins were restored to nearly wild-type levels in these cells, confirming that their amounts in resting cells are maintained through ATG5/autophagy.

### Effect of ATG5 deficiency on immune responses to viral and bacterial PAMPs.

**FIG 7:**
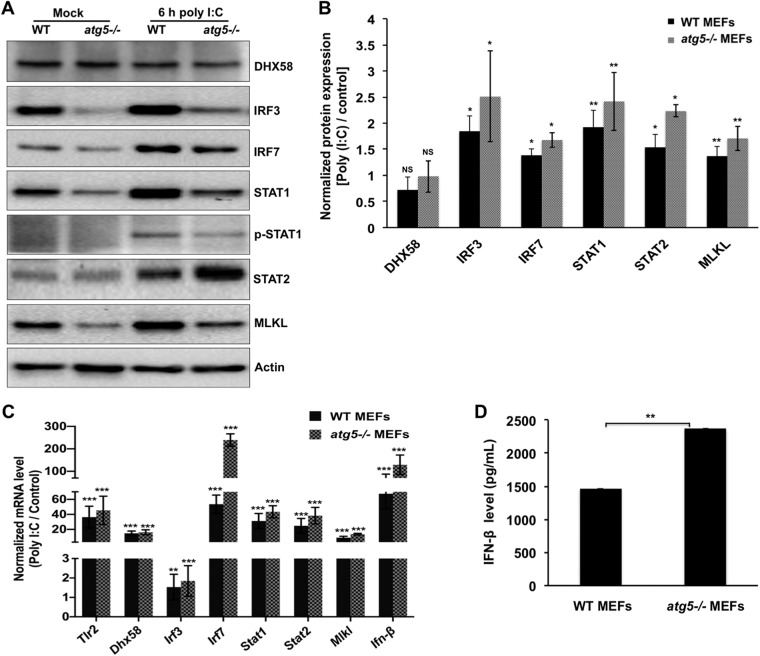
ATG5-deficient MEFs show an enhanced type I interferon response to the dsRNA mimic poly(I·C). WT and *atg5^−/−^* MEFs were transfected with 1 μg/ml poly(I·C) for 6 h or mock transfected. (A) Western blot showing the levels of DHX58, IRF3, IRF7, STAT1, pSTAT1, STAT2, MLKL, and actin (loading control). (B) Bar graph showing normalized protein expression [poly(I·C)/control] of the indicated proteins in WT and *atg5^−/−^* MEFs. Densitometry analysis was performed using ImageJ software. (C) mRNA levels of various immune effector genes, including *Dhx58*, *Irf3*, *Irf7*, *Stat1*, *Stat2*, *Mlkl*, and *Ifn*β, were quantitated by qRT-PCR. (D) The supernatant collected from treated cells was used to perform an ELISA for IFN-β. For panels B to D, data presented are means ± standard deviations of values obtained from 3 independent experiments. Student’s *t* test was used to calculate *P* values (*, *P < *0.05; **, *P < *0.01; ***, *P < *0.001).

We next assessed the activation of immune effectors in both WT and *atg5^−/−^* MEFs in response to stimulation with poly(I·C) (synthetic double-stranded RNA [dsRNA] virus analog). Recognition of dsRNA activates IRF3-dependent expression of antiviral factors. By 6 h posttreatment, poly(I·C) significantly upregulated the levels of IRF3, IRF7, STAT1, STAT2, and MLKL in both cell types (Fig. 7A and B). Despite similar activation, the protein levels of these effectors were still lower under autophagy-deficient conditions than for the control (Fig. 6A and B). Since *atg5^−/−^*MEFs have lower STAT1 levels, the level of STAT1 phosphorylation in these cells was also lower (Fig. 7A). However, significant transcriptional activation of all these genes and *IFN*β was observed in these cells, with *Irf7* and *IFN*β showing severalfold-higher levels of upregulation in *atg5^−/−^*MEFs than in WT MEFs (Fig. 7C). In accordance with higher RNA levels, the level of secretion of IFN-β in these cells was also higher (Fig. 7D). Enhanced IFN and cytokine production in *atg5^−/−^* MEFs in response to vesicular stomatitis virus (VSV) infection and poly(I·C) has been reported in previous studies, based on association of the ATG5-ATG12 complex with RIG-1, MDA-5, and IPS-1 (56), and by enhanced reactive oxygen species (ROS) production mediated by dysfunctional mitochondria (57). Another recent study demonstrated that in response to influenza A virus infection, ATG5-deficient cells had higher IFN-β expression levels than autophagy-competent cells (17).

**FIG 8:**
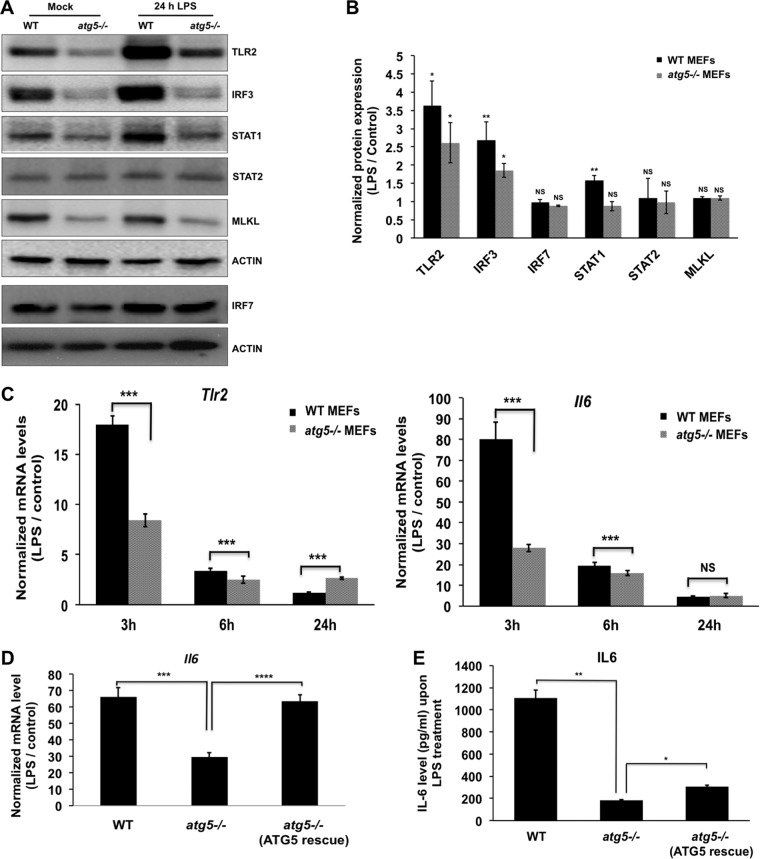
ATG5-deficient MEFs show reduced immune responses to the bacterial PAMP LPS. WT and *atg5^−/−^* MEFs were treated with 100 ng/ml LPS. (A) Western blot showing the levels of TLR2, IRF3, IRF7, STAT1, STAT2, MLKL, and actin (loading control) 24 h after LPS treatment. (B) Bar graph showing normalized protein expression (LPS/control) of the indicated proteins in WT and *atg5^−/−^* MEFs. Densitometry analysis was performed using ImageJ software. (C) Normalized mRNA levels of *Tlr2* and *Il-6* 3, 6, and 24 h after LPS treatment. (D and E) LPS treatment was given for 3 h to WT MEFs (vector transfected), *atg5^−/−^* MEFs (vector transfected), and *atg5^−/−^* MEFs (pCI-neo-mApg5 transfected). Transcript levels of *Il-6* (D) and secreted IL-6 in the culture supernatant (E) were estimated. Data presented are means ± standard deviations of values obtained from 3 independent experiments. Student’s *t* test (B and C) or ANOVA followed by Dunnett’s *post hoc* comparison test (D and E) was used to calculate *P* values (*, *P < *0.05; **, *P < *0.01; ***, *P < *0.001; ****, *P < *0.0001).

We further checked for the capacity of the *atg5^−/−^* MEFs to mount an immune response against the common bacterial PAMP LPS. A 24-h treatment with LPS resulted in significant increases in levels of TLR2 and IRF3 in both cell types (Fig. 8A and B). A comparison of the transcriptional levels of *Tlr2* and *Il-6* revealed that the *atg5^−/−^* MEFs were suppressed for activation at early times (3 and 6 h) after LPS treatment (Fig. 8C). To check if the transcriptional activation and secretion of the major proinflammatory cytokine interleukin-6 (IL-6) were dependent on ATG5, we expressed ATG5 in autophagy-deficient cells. After 3 h of LPS treatment, we observed significant recovery of *Il-6* mRNA levels and IL-6 secretion in ATG5-rescued cells (Fig. 8D and E).

**FIG 9:**
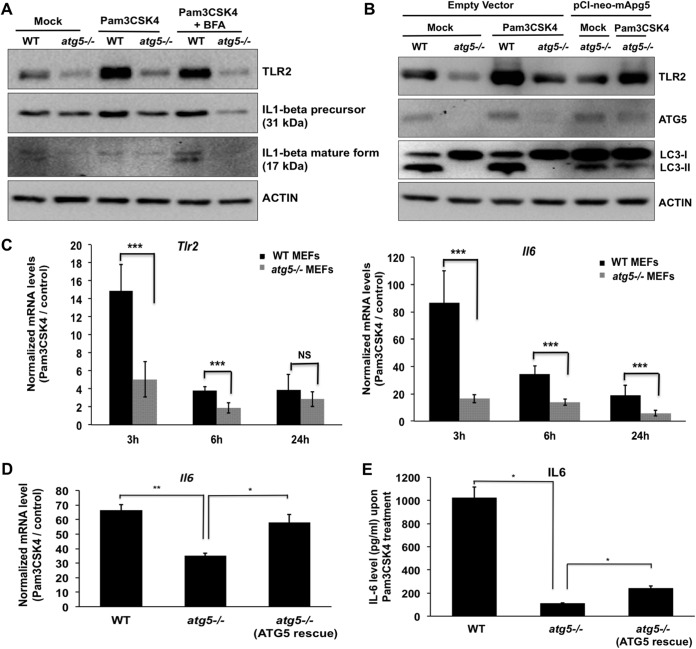
ATG5-deficient MEFs show reduced responses to the TLR2 agonist Pam3CSK4, which is rescued by ATG5 reexpression. (A) WT and *atg5^−/−^* MEFs were treated with 100 ng/ml Pam3CSK4 for 6 h. A total of 1 μg/ml brefeldin A (BFA) was added 4 h before harvest to block protein secretion. Western blots show the levels of TLR2, IL-1β, and actin (loading control). (B) WT and *atg5^−/−^* MEFs were transfected with an empty vector or pCI-neo-mApg5 and treated with Pam3CSK4 for 3 h. Cell lysates were blotted for TLR2, ATG5, LC3, and actin (loading control). (C) Transcript levels of *Tlr2* and *Il-6* 3, 6, and 24 h after Pam3CSK4 treatment. (D and E) Pam3CSK4 treatment was given for 3 h to WT MEFs (vector transfected), *atg5^−/−^* MEFs (vector transfected), and *atg5^−/−^* MEFs (pCI-neo-mApg5 transfected). Transcript levels of *Il-6* (D) and secreted IL-6 in the culture supernatant (E) were estimated. Data presented are means ± standard deviations of values obtained from 3 independent experiments. Student’s *t* test (B and C) or ANOVA followed by Dunnett’s *post hoc* comparison test (D and E) was used to calculate *P* values (*, *P < *0.05; **, *P < *0.01; ***, *P < *0.001).

A striking feature of *atg5^−/−^* MEFs was lower levels of TLR2, a member of the Toll-like receptor signaling family. TLR2 forms heterodimeric complexes with either TLR1 or TLR6 and binds to diacylated and triacylated lipopeptides (58). The TLR2/1 heterodimer recognizes the triacylated lipopeptide Pam3CSK4 (59). We further characterized the effect of ATG5/autophagy on TLR2 and its downstream signaling by using its specific agonist Pam3CSK4 (Fig. 9). Samples prepared from mock- and Pam3CSK4-treated WT and *atg5^−/−^* MEFs were treated with brefeldin A to block protein secretion and analyzed by Western blotting (Fig. 9A). We observed that the absence of autophagy reduced IL-1β synthesis and secretion and *Il-6* activation in response to TLR2 activation (Fig. 9A and C). We further checked if restoration of ATG5 expression in the knockout (KO) MEFs would have any effect on Pam3CSK4-induced signaling through TLR2 (Fig. 9B, D, and E). The protein levels of TLR2 (Fig. 9B), *Il-6* activation (Fig. 9D), and IL-6 secretion (Fig. 9E) were recovered by ATG5 expression in the autophagy-deficient cells. Collectively, out data indicate a crucial role of functional autophagy/ATG5 for activation of TLR2 signaling and IL-6 production.

## DISCUSSION

This study serves as a resource for understanding the impact of autophagy deficiency on the basal fibroblast proteome. Our data show that autophagy modulates nearly 14% of the cellular proteome, indicating its role in a plethora of cellular functions and in maintaining intracellular homeostasis.

While autophagy plays a critical role under stress conditions, such as starvation and pathogen infection, basal autophagy is also essential for constitutive turnover of cellular contents. The role of autophagy in protein turnover was recently quantified in a study by comparing protein half-lives in autophagy-deficient primary human fibroblasts, using time-resolved isotopic labeling and mass spectrometry. This study showed that the proteasome and the CCT/TriC chaperonin are autophagy substrates and are stabilized under autophagy-deficient conditions (60). This highlighted how autophagy can impact protein degradation through the ubiquitin proteasomal pathway. A subsequent study by the same group showed enhanced degradation of long-lived proteins in quiescent fibroblasts via upregulation of autophagy (61). Autophagy has also been shown to be a critical player in the balance between senescence and apoptosis (62). Studies in *Arabidopsis* have shown that *atg5* mutants accumulate proteins but also have higher activities of the proteasome and papain-like cysteine protease, which could contribute to enhanced cell death (63). These studies have given mechanistic insights into how autophagy modulates basal protein levels in different experimental systems.

Our study with MEFs demonstrates that autophagy regulates proteins of diverse biological processes, ranging from development, metabolism, cell adhesion, and transport to innate and adaptive immunity. Changes in the basal levels of these proteins can potentially rewire entire cellular pathways and influence biological outcomes. Significantly, the lysosomal compartment was impacted by ATG5 loss, as seen by reduced levels of several lysosomal proteins (LAMP1 and LAMP2), enzymes, and vacuolar ATPases. Several upregulated proteins in the autophagy-deficient cells were receptors with an extensive range of functions (TGF-β signaling, JAK-STAT signaling, and cytokine and interferon receptors). Several of these receptors signal at the plasma membrane by binding to their ligands and are internalized into endosomes for recycling or are degraded by targeting to the late endosome/lysosome. Higher levels of these receptors are indicative of the role of autophagy in their turnover and the potential to impact the strength of the signal emanating from them. Some of these receptors are known to be modulated by autophagy, while others (ACVR1A, AVCR2A, JAMA, JAMC, and SDC2) have been described for the first time in this study. We have analyzed all the upregulated proteins for potential LIR motifs (24) and observed that 63% of these were potential autophagy substrates. A recent study described a new class of ATG8-interacting proteins that utilize a ubiquitin-interacting motif-like sequence for high-affinity binding (64).

Immune-related proteins formed a major subgroup that was dysregulated upon ATG5 deficiency. While several cytokine and interferon receptors were upregulated in these cells, key immune sensors (TLR2) and effectors (IRF3, IRF7, MLKL, and STAT1/3/5/6) had lower levels. Their expression in the KO MEFs was restored by the expression of ATG5, validating the essential role of ATG5/autophagy in modulating their levels in resting cells.

We observed that protein levels of IRF3 were low, while its transcript level was upregulated in *atg5^−/−^* MEFs. However, Baf A_1_ treatment of WT MEFs confirmed that basal levels of IRF3 were directly modulated by autophagy. This suggests that in ATG5-deficient cells, either IRF3 stabilization factors are lacking or there is enhanced proteasomal degradation of IRF3. The E3 ubiquitin ligase c-Cbl has been shown to be a negative regulator of IRF3 stability, promoting its polyubiquitination and proteasomal degradation (65). Studies have also shown that IRF3 is constitutively associated with IFITM3, which regulates IRF3 homeostasis by mediating its autophagic degradation (66).

Several studies have dissected the role of autophagy in the context of diverse infections in different cell types. Autophagy has been shown to modulate different steps of immune signaling during bacterial and viral infections (1). It can either enhance the immune response by facilitating PAMP recognition by TLRs present on autophagosomes or suppress signaling by degrading various immune-related proteins in autolysosomes (67–69). Autophagy is involved in limiting the type I IFN response by (i) clearing the RIG-I pathway proteins in autophagy-deficient Ras-driven cancer cells (70), (ii) degrading NF-κB signaling components (71) and IRF3 by TRIM21-mediated selective degradation, and (iii) interfering with RLR-IPS1 interaction-driven downstream signaling.

Our study demonstrates dysregulation of critical immune receptors, signaling proteins, and effectors under ATG5 deficiency. The role of autophagy in determining the outcome of the immune response is likely to be highly complex and context dependent, as immune responses are likely to have multilayered regulation, at the receptor, adaptor, transcriptional, translational, and/or posttranslational level. Consistent with previous studies (56, 57), we observed that *Atg5* KO cells can mount a higher type I IFN response to the dsRNA mimic poly(I·C).

Significantly lower levels of TLR2 were seen in the *atg5^−/−^* MEFs, which were rescued by ATG5 reexpression. IL-6 activation and secretion in response to LPS and the TLR2 ligand Pam3CSK4 were also directly modulated by autophagy, as reexpression of ATG5 was able to rescue the stunted inflammatory response under autophagy-deficient conditions. TLR2 signaling is closely related to autophagy induction and flux (72) and plays important roles in innate immune responses to several bacterial infections, including Staphylococcus aureus, Pseudomonas aeruginosa, Klebsiella pneumoniae, and Mycobacterium tuberculosis infections (73, 74), and in the regulation of inflammation (75).

Our data and analyses provide a global profile of how autophagy modulates the fibroblast cellular proteome and can potentially impact key cellular processes. We also validate the crucial role of autophagy in modulation of innate immune responses. This study should serve as a useful resource for the molecular and cellular machinery that is regulated by autophagy/ATG5.

## MATERIALS AND METHODS

### Cells and reagents.

WT and *Atg5*-deficient (*atg5^−/−^*) mouse embryonic fibroblasts were a kind gift from Noboru Mizushima and obtained through the RIKEN Bio-Resource Cell Bank (catalog numbers RCB2710 and RCB2711). MEFs were grown in Dulbecco’s modified Eagle’s medium (DMEM) (catalog number AL007A; HiMedia) supplemented with 10% fetal bovine serum (FBS) (catalog number RM10432; HiMedia). All media were additionally supplemented with 100 μg/ml penicillin-streptomycin and 2 mM l-glutamine. Antibodies and reagents used in this study are listed in Table S1 in the supplemental material.

10.1128/mSystems.00481-19.5 TABLE S1 List of antibodies/reagents and their sources. Download Table S1, DOCX file, 0.1 MB. Copyright © 2019 Sharma et al. 2019 Sharma et al. This content is distributed under the terms of the Creative Commons Attribution 4.0 International license.

### TMT-based mass spectrometry.

**(i) Sample preparation.** The experimental system was designed for 8-plex TMT-based mass spectrometry analysis (Fig. S1A). For this study, we analyzed four samples, which were two biological replicates each of WT and *atg5^−/−^* MEFs. Cells grown in conditioned DMEM were lysed in a buffer consisting of 50 mM Tris (pH 8.5), 8 M urea, 1% SDS, and protease and phosphatase inhibitors (catalog number 05056489001; Roche). Protein quantification was done using a micro-bicinchoninic acid (BCA) assay (catalog number 23225; Pierce). One milligram of protein for each sample was precipitated using a methanol-chloroform precipitation method and sent for TMT-based mass spectrometry to the Thermo Fisher Center for Multiplexed Proteomics (TCMP) facility at Harvard Medical School. Sample digestion, TMT labeling, TMT-based mass spectrometry, and data searches were performed at the TCMP facility. After protein recovery, samples were quantitated, immediately reduced with dithiothreitol (DTT), and alkylated with iodoacetamide (IAM). Protein from each sample was digested using endoproteinase-LysC and trypsin, and 75 μg of the peptides of each sample was labeled with TMT reagent. Peptides thus generated were fractionated using basic-pH reverse-phase (bRP) columns.

**(ii) Mass spectrometry data acquisition.** Peptide fractions from HPRP (high-pH reverse-phase) separation were analyzed on an Orbitrap fusion mass spectrometer. Peptides were separated using a gradient of 3% to 25% acetonitrile in 0.125% formic acid over 180 min. Peptides were detected (MS1) and quantified (MS3) in the Orbitrap, and peptides were sequenced (MS2) in the ion trap.

**(iii) Database search and quantification.** The raw files were searched using the Sequest algorithm against the UniProt Mus musculus proteome FASTA database. The FASTA database also contained the reversed sequences as decoys and known contaminants. TMT tags and carbamidomethylation were used as fixed modifications, and methionine oxidation was used as a variable modification. The peptide identifications were filtered at a false discovery rate (FDR) of 1% using the concatenated target-decoy strategy combined with linear discriminant analysis. An FDR of 1% was applied to the proteins from 4 fractions, and quantitation was carried out from peptides with a summed signal-to-noise (S/N) threshold of ≥200 and an isolation specificity of 0.5.

At a 1% FDR, 151,284 peptides were identified, corresponding to 10,032 proteins. Of these, 8,745 proteins were quantified using TMT reporter tags. The TMT intensities obtained were normalized, and the replicate intensities were averaged, to calculate the combined areas. These combined replicate areas were used to calculate the relative ratios. Proteins identified with fewer than two unique peptides were excluded. Further analysis was performed with 7,795 proteins that were identified with more than two unique peptides. The fold change was calculated for *atg5^−/−^* cells versus the WT as follows: FC = *Atg5* KO/WT. Three different stringency threshold conditions were applied to the data: low (FC of ≥1.5 and FC of ≤0.666), medium (FC of ≥2 and FC of ≤0.5) and high (FC of ≥3 and FC of ≤0.33). The numbers and identities of over- or underexpressed proteins observed under each condition are provided in Data Set S1. To ensure maximum coverage and to avoid the loss of information for proteins that might show smaller variations in their levels under autophagy deficiency, we utilized differentially expressed proteins under low-stringency conditions for detailed analysis.

### Gene ontology (GO) analysis.

The proteins observed to be underexpressed or overexpressed were used separately for a functional enrichment analysis for biological processes, molecular functions, and cellular components using GeneCodis 3.0 (76). KEGG pathways were analyzed using GeneCodis, and Reactome pathway enrichment was carried out using MetaScape (77). All the functional enrichment categories were filtered at a corrected *P* value of <0.05 (hypergeometric test in GeneCodis). For pathway analysis, the filtered list based on the *P* value from MetaScape and GeneCodis is unified based on the proteins present in each category for a combined analysis.

### Protein-protein interaction network analysis.

For protein-protein interaction (PPI) network enrichment analysis, the lists of differentially expressed proteins under the two conditions were studied using the STRING Web server. The interaction network was made at a medium confidence of 0.400, with evidence present via experiments, databases, and coexpression only. The network was analyzed based on KEGG pathway enrichment analysis done with the STRING functional enrichment tool.

### Western blotting.

Mock-treated and treated WT and *atg5^−/−^* MEFs were washed using phosphate-buffered saline (PBS) and lysed by adding cell lysis buffer (50 mM Tris-HCl [pH 7.5], 150 mM NaCl, 1% Triton X-100, a protease inhibitor cocktail [catalog number P8340; Sigma], and phenylmethylsulfonyl fluoride [PMSF] [CAS number 329-98-6; Sigma]). Protein estimation was done using the micro-BCA assay (catalog number 23225; Pierce). The lysate was mixed with 4× Laemmli buffer (6% SDS, 40% glycerol, 0.04% bromophenol blue, 20% β-mercaptoethanol, 0.25 M Tris [pH 6.8], and water) and further boiled for 10 min at 95°C. Equal amounts of cellular protein were loaded onto and separated on polyacrylamide gels and later transferred to a polyvinylidene difluoride (PVDF) membrane (Immobilon-P, catalog number IPVH00010; Merck Millipore) for immunoblotting. Band intensities were quantitated by using ImageJ software. Data are presented as mean values ± standard deviations (SD) obtained from 3 independent experiments.

### Expression of ATG5 in *atg5^−/−^* MEFs.

The vector backbone or plasmid pCI-neo-mApg5 (Addgene plasmid 22956) (14) was transfected into WT and *atg5^−/−^* MEFs, respectively, using Amaxa cell line Nucleofector kit V according to the manufacturer’s manual (catalog number VCA-1003; Lonza). At 24 h posttransfection, cells were treated with 100 ng/ml of LPS (catalog number L2630; Sigma) or Pam3CSK4 (catalog number tlrl-pms; InvivoGen) for 3 h. Cells were lysed for Western blotting, cellular RNA was extracted for real-time reverse transcription-quantitative PCR (qRT-PCR), and the culture supernatant was used for an IL-6 enzyme-linked immunosorbent assay (ELISA).

### Real-time reverse transcription-quantitative PCR.

Total RNA from cells was extracted by lysis in RNAiso Plus reagent (TaKaRa), and 500 ng of total RNA was used for cDNA preparation using random hexamers and the ImProm-II reverse transcription system (Promega). Primers for all the genes were designed based on sequences available in the Harvard quantitative PCR (qPCR) primer bank. All qPCRs were performed using 2× SYBR green reagent (TaKaRa) in a QuantStudio 6 flex RT-PCR machine. *Gapdh* levels were used as the internal housekeeping control. The PCR conditions were as follows: 94°C for 2 min (1 cycle) and 94°C for 15 s, 55°C for 30 s, and 72°C for 1 min (40 cycles). All experiments had biological duplicates and were performed independently three or more times. The fold changes in the expression levels of genes are presented as means ± SD of data from three or more independent experiments. Primer sequences used for quantification of various genes are provided in Table S2.

10.1128/mSystems.00481-19.6 TABLE S2 List of primers used in the study. Download Table S2, DOCX file, 0.1 MB. Copyright © 2019 Sharma et al. 2019 Sharma et al. This content is distributed under the terms of the Creative Commons Attribution 4.0 International license.

### Interferon/IL-6 ELISA.

The culture supernatant was collected from the cells that were mock transfected or poly(I·C) (catalog number P1530; Sigma) transfected for 6 h, mock/LPS/Pam3CSK4 treated for 3 h, and centrifuged to remove any debris. An ELISA was performed according to the manufacturers’ protocols for IFN-β (catalog number 42400; PBL Assay Science) and IL-6 (catalog number DY406-05; R&D Systems), respectively.

### Statistical analysis.

Statistical analysis was done using unpaired Student’s *t* test or one-way analysis of variance (ANOVA) followed by Dunnett’s *post hoc* comparison test. Differences were considered significant at *P* values of <0.05, 0.01, 0.001, and <0.0001, as indicated in the figures. Error bars indicate means ± SD (*n* = 3).

### Data availability.

The mass spectrometry proteomics data have been deposited in the ProteomeXchange Consortium via the PRIDE (78) partner repository under data set accession number PXD014986.
