# Supplementary figures and images for: Quantitative Proteome Analysis of Atg5-Deficient Mouse Embryonic Fibroblasts Reveals the Range of the Autophagy-Modulated Basal Cellular Proteome
Source: mSystems. 2019 Nov 5;4(6):e00481-19. doi: 10.1128/mSystems.00481-19 (PMC6832020; doi:10.1128/mSystems.00481-19)

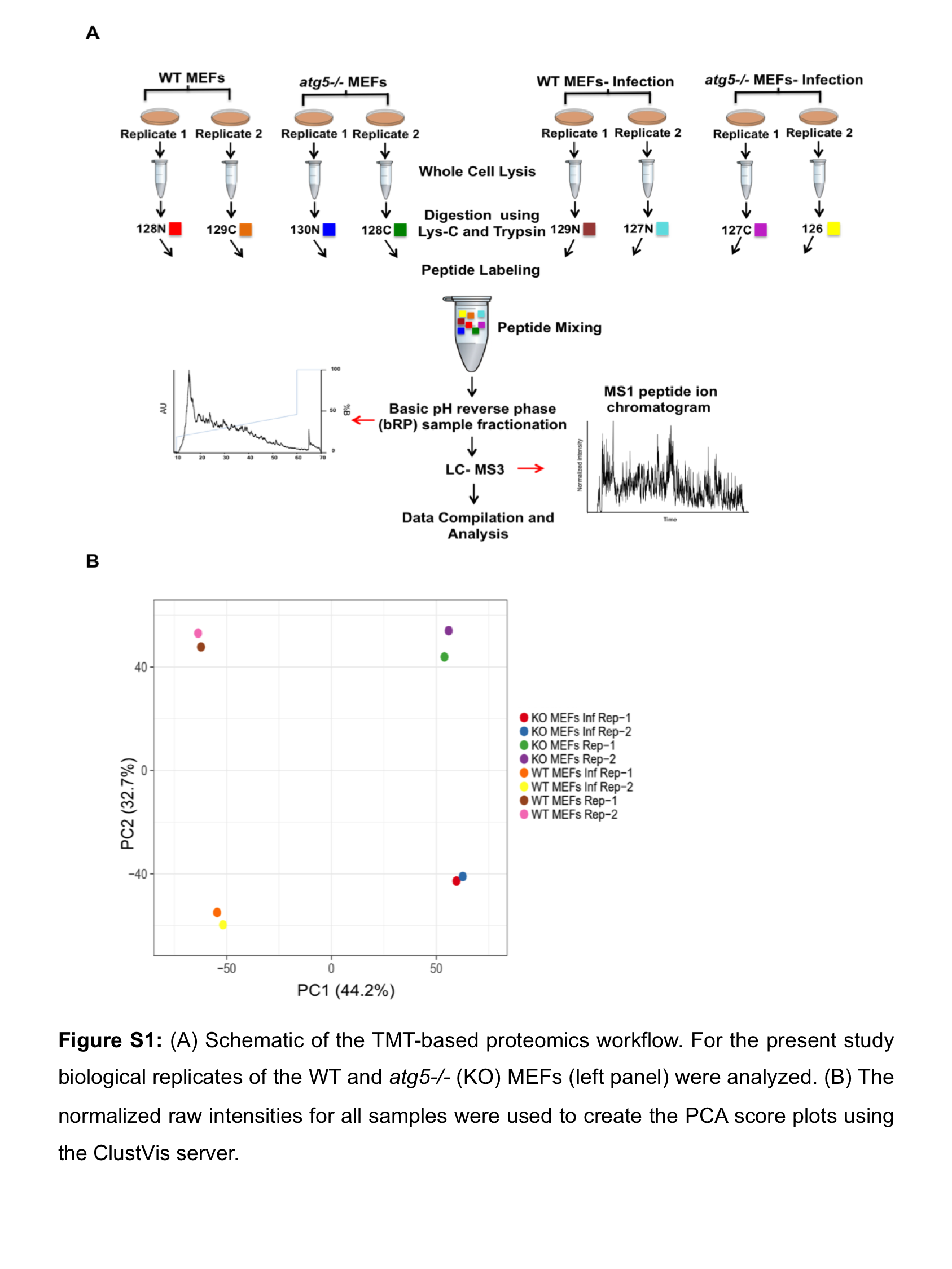

Supplement: FIG S1 [file mSystems.00481-19-sf001.tif]

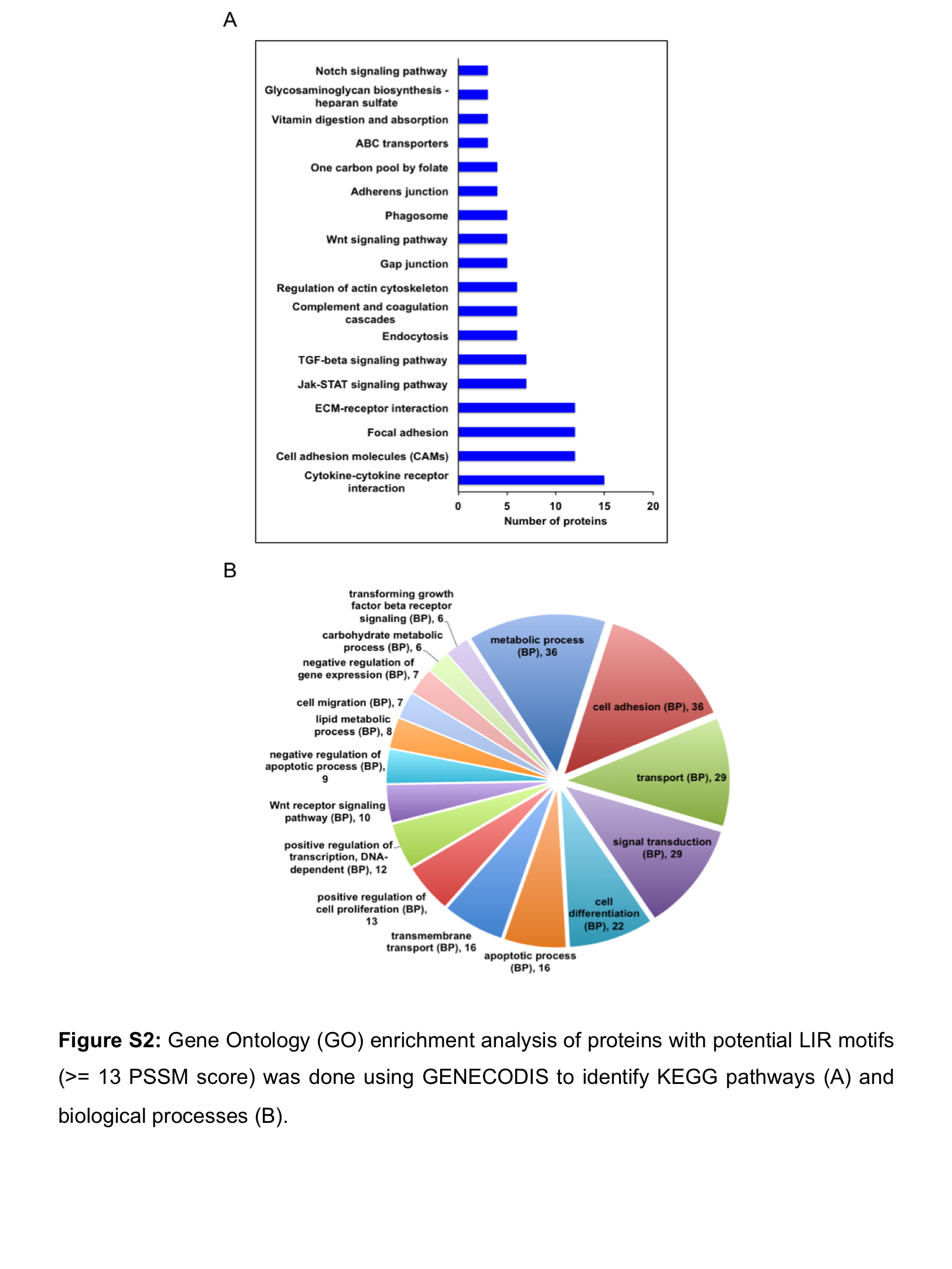

Supplement: FIG S2 [file mSystems.00481-19-sf002.tif]

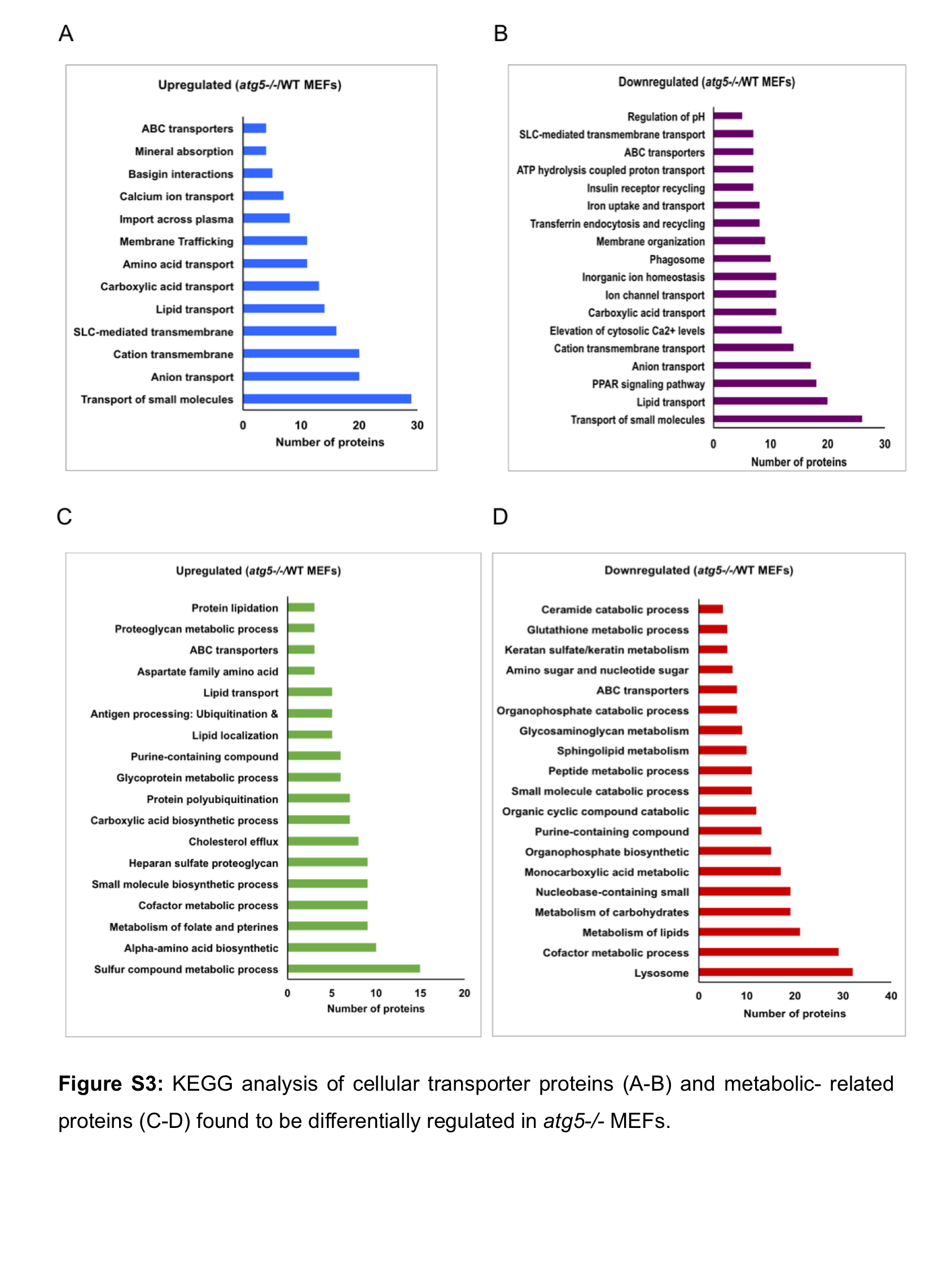

Supplement: FIG S3 [file mSystems.00481-19-sf003.tif]

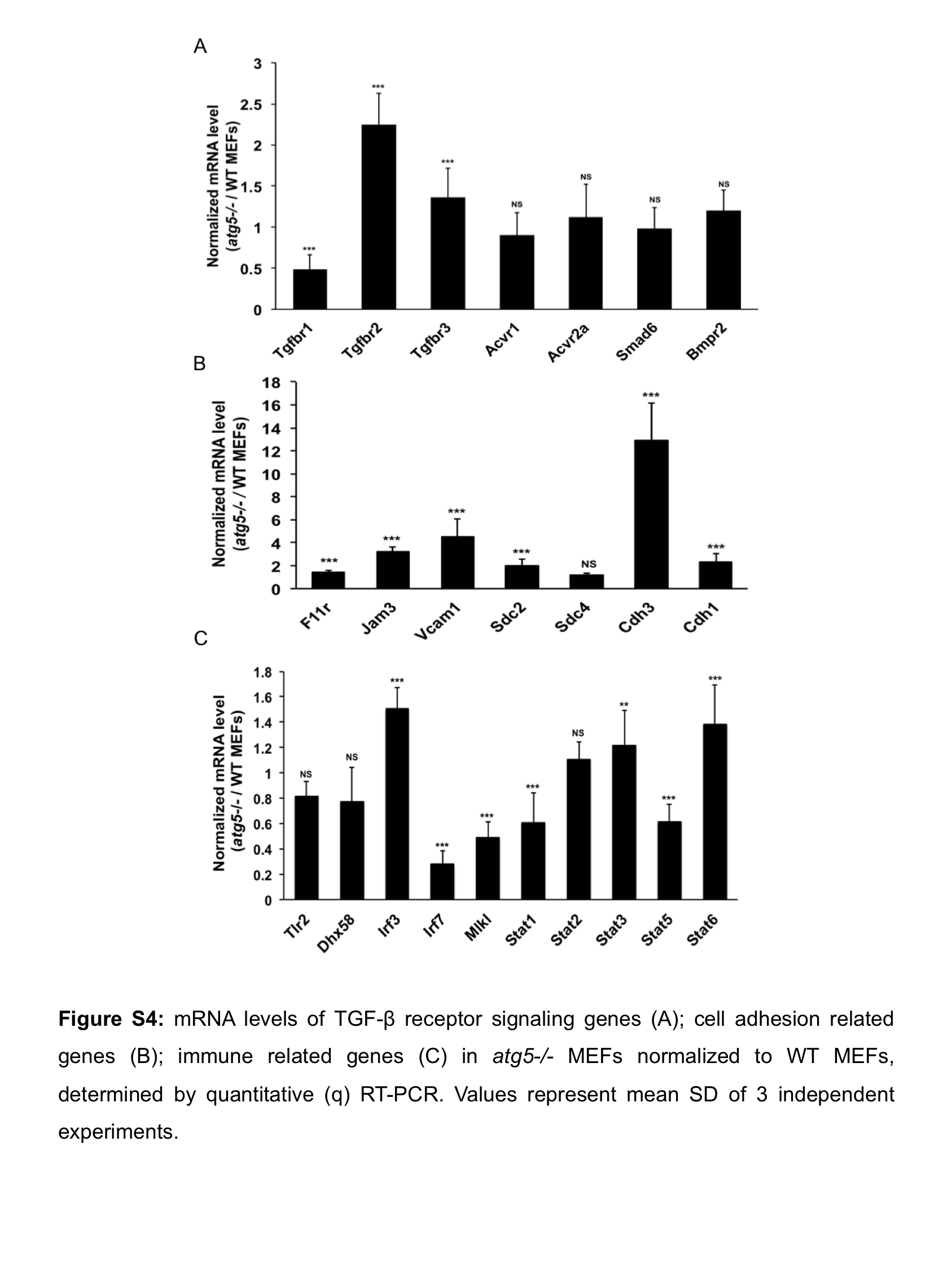

Supplement: FIG S4 [file mSystems.00481-19-sf004.tif]
