# Supplementary material for: Quantitative Proteome Analysis of Atg5-Deficient Mouse Embryonic Fibroblasts Reveals the Range of the Autophagy-Modulated Basal Cellular Proteome
Source: mSystems. 2019 Nov 5;4(6):e00481-19. doi: 10.1128/mSystems.00481-19 (PMC6832020; doi:10.1128/mSystems.00481-19)
Supplement: TABLE S1 [file mSystems.00481-19-st001.docx]

**Table S1: List of antibodies/reagents and their source**

| ANTIBODIES/Reagents | SOURCE | IDENTIFIER |
| --- | --- | --- |
| ACTIN | Santa Cruz Biotechnology | Cat #: sc-47778 |
| ACVR1 | Santa Cruz Biotechnology | Cat #: sc-374523 |
| ACVR2a | Santa Cruz Biotechnology | Cat #: sc-515826 |
| ATG5 | Cell Signaling | Cat #: 12994s |
| CADHERIN-1 | Cell Signaling | Cat #: 3195S |
| CAHERIN-3 | Santa Cruz Biotechnology | Cat #: sc-514481 |
| DHX58 | Abcam | Cat #: ab67270 |
| IL1β | Abcam | Cat #: ab9722 |
| IRF3 | Abcam | Cat #: ab68481 |
| IRF7 | Abcam | Cat #: ab109255 |
| JAM-1 | Santa Cruz Biotechnology | Cat #: sc-53623 |
| JAM-3 | Signalway antibody | Cat #: signalway-45639-2 |
| LC-3B | Cell Signaling | Cat #: 3868 |
| MLKL | Abcam | Cat #: ab172868 |
| SMAD6 | Santa Cruz Biotechnology | Cat #: sc-25321 |
| STAT1 | Cell Signaling | Cat #: 9172 |
| STAT2 | Cell Signaling | Cat #: 72604S |
| STAT3 | Cell Signaling | Cat #: 4904 |
| STAT5 | Cell Signaling | Cat #: 94205 |
| STAT6 | Cell Signaling | Cat #: 5397 |
| SYNDECAN2 | Invitrogen | Cat #: 366200 |
| TGFβR-I | Santa Cruz Biotechnology | Cat #: sc-101574 |
| TGFβR-II | Santa Cruz Biotechnology | Cat #: sc-17799 |
| TGFβR-III | Santa Cruz Biotechnology | Cat #: sc-74511 |
| TLR2 | Abcam | Cat #: ab209217 |
