# Supplementary material for: Quantitative Proteome Analysis of Atg5-Deficient Mouse Embryonic Fibroblasts Reveals the Range of the Autophagy-Modulated Basal Cellular Proteome
Source: mSystems. 2019 Nov 5;4(6):e00481-19. doi: 10.1128/mSystems.00481-19 (PMC6832020; doi:10.1128/mSystems.00481-19)
Supplement: TABLE S2 [file mSystems.00481-19-st002.docx]

**Table S2: List of primers used in the study**

| **GENE NAME (Mouse)** | **PRIMER SEQUENCE (5'-3')** |
| --- | --- |
| *Acvr1-*F | CATCGCTTCAGACATGACCTC |
| *Acvr1-*R | CTAACCGTATCCAGAGTAGTG |
| *Acvr2a-* F | CCCTCCTGTACTTGTTCCTACTCA |
| *Acvr2a-* R | GCAATGGCTTCAACCCTAGT |
| *Bmpr2-*F | GAGCCCTCCCTTGACCTG |
| *Bmpr2-*R | GTATCGACCCCGTCCAATC |
| *Cdh1-*F | CAGGTCTCCTCATGGCTTTGC |
| *Cdh1-*R | CTTCCGAAAAGAAGGCTGTCC |
| *Cdh3-F* | CTGGAGCCGAGCCAAGTTC |
| *Cdh3-R* | GGAGTGCATCGCATCCTTCC |
| *Dhx58*-F | GGAAGTGATCTTACCTGCTCTGG |
| *Dhx58-*R | TTGCCTCTGTCTACCGTCTCT |
| *F11r -*F | TCTCTTCACGTCTATGATCCTGG |
| *F11r- R* | TTTGATGGACTCGTTCTCGGG |
| *Gapdh-F* | CGTCCCGTAGACAAAATGGT |
| *Gapdh-R* | TTGATGGCAACAATCTCCAC |
| *Il-6-*F | CCA CGG CCT TCC CTA CTT C |
| *Il-6-*R | TTG GGA GTG GTA TCC TCT GTG A |
| *Irf3*-F | GAGAGCCGAACGAGGTTCAG |
| *Irf3*-R | CTTCCAGGTTGACACGTCCG |
| *Irf7-*F | GAGACTGGCTATTGGGGGAG |
| *Irf7-*R | GACCGAAATGCTTCCAGGG |
| *Jam3-*F | CTGCGACTTCGACTGTACG |
| *Jam3-*R | TTCGGTTGCTGGATTTGAGATT |
| *Mlkl-*F | GGATTGCCCTGAGTTGTTGC ′ |
| *Mlkl-R* | AACCGCAGACAGTCTCTCCA |
| *Nfkβ-F* | ATGGCAGACGATGATCCCTAC |
| *Nfkβ-*R | TGTTGACAGTGGTATTTCTGGTG |
| *Sdc2-*F | TGTGTCCGCAGAGACGAGAA |
| *Sdc2-*R | GGAATCAGTTGGGATGTTGTCA |
| *Sdc4-*F | TTTGCCGTTTTCCTGATCCTG |
| *Sdc4-R* | TTGCCCAAGTCGTAACTGCC |
| *Smad6-* F | ATTCTCGGCTGTCTCCTCCT |
| *Smad6- R* | CCCTGAGGTAGGTCGTAGAA |
| *Stat1*-F | TCACAGTGGTTCGAGCTTCAG |
| *Stat1*-R | GCAAACGAGACATCATAGGCA |
| *Stat2-* F | TCCTGCCAATGGACGTTCG |
| *Stat2-* R | GTCCCACTGGTTCAGTTGGT |
| *Stat3-*F | AATGGAAATTGCCCGGATC |
| *Stat3-*R | AGGCGAGACTCTTCCCACAG |
| *Stat5-*F | CGCTGGACTCCATGCTTCTC |
| *Stat5-*R | GACGTGGGCTCCTTACACTGA |
| *Stat6-*F | CTGGGGTGGTTTCCTCTTG |
| *Stat6-*R | TGCCCGGTCTCACCTAACTA |
| *Tgfbr1-*F | CAACCCAGGTCCTTCCTAAA |
| *Tgfbr1-*R | GGAGAGCCCTGGATACCAAC |
| *Tgfbr2-*F | AGATGGCTCGCTGAACACTACCAA |
| *Tgfbr2-R* | AGAATCCTGCTGCCTCTGGTCTTT |
| *Tgfbr3-*F | CCTCCTCCACAGATTTTCCA |
| *Tgfbr3-*R | CCCAGATCAAGCCTTCTGAG |
| *Tlr2*-F | GCAAACGCTGTTCTGCTCAG |
| Tlr2-R | AGGCGTCTCCCTCTATTGTATT |
| *TNFα-* F | CAT CTT CTC AAA ATT CGA GTG ACA A |
| *TNFα-* R | TGG GAG TAG ACA AGG TAC AAC CC |
| *Vcam1-*F | AGTTGGGGATTCGGTTGTTCT |
| *Vcam1-*R | CCCCTCATTCCTTACCACCC |
